# Oxygen–Glucose Deprivation Increases NR4A1 Expression and Promotes Its Extranuclear Translocation in Mouse Astrocytes

**DOI:** 10.3390/brainsci14030244

**Published:** 2024-02-29

**Authors:** Kengo Moriyama, Asako Horino, Kuniko Kohyama, Yasumasa Nishito, Tomohiro Morio, Hiroshi Sakuma

**Affiliations:** 1 Department of Brain and Neuroscience, Tokyo Metropolitan Institute of Medical Science, Tokyo 156-8506, Japan; moriyama-kg@igakuken.or.jp (K.M.);; 2 Department of Pediatrics, Tokyo Medical and Dental University, Tokyo 113-8510, Japan; 3 Technology Research Division, Tokyo Metropolitan Institute of Medical Science, Tokyo 156-8506, Japan

**Keywords:** HIF-1α, NR4A1, NR4A3, pexidartinib, oxygen–glucose deprivation, mitochondria, reoxygenation, CDIM8

## Abstract

Hypoxic–ischemic brain injury induces metabolic dysfunction that ultimately leads to neuronal cell death. Astrocytes, a type of glial cell, play a key role in brain metabolism; however, their response to hypoxic–ischemic brain injury is not fully understood. Microglia were removed from murine primary mixed glial cultures to enrich astrocytes. Next, we explored genes whose expression is altered following oxygen–glucose deprivation using a microarray. Microarray analysis revealed that the expression of *Nr4a1* and *Nr4a3* is markedly increased in astrocyte-enriched cultures after 15 h of oxygen–glucose deprivation. The expression of both *Nr4a1* and *Nr4a3* was regulated by HIF-1α. At the protein level, NR4A1 was translocated from the nucleus to the cytoplasm following oxygen–glucose deprivation and co-localized with mitochondria in apoptotic cells; however, its localization was restored to the nucleus after reoxygenation. Oxygen–glucose deprivation causes an increase in NR4A1 mRNA in astrocytes as well as its nuclear to cytoplasmic transfer. Furthermore, reoxygenation enhances NR4A1 transcription and promotes its nuclear translocation.

## 1. Introduction

Perinatal hypoxic–ischemic encephalopathy (HIE) is associated with high neonatal mortality and severe long-term neurological morbidity [1,2], with a frequency of 1–8 per 1000 live births in developed countries [3,4]. Transient oxygen deprivation and ischemia are recognized as major causes of HIE; while initial energy failure and reperfusion can restore cellular energy metabolism [5,6], neuron and glial cell loss of varying degrees is inevitable [6]. More importantly, hypoxia/ischemia impair the entry of Ca^2+^ ions into cells and promote free radical and reactive oxygen species production, excitotoxic amino acid release, inflammation, and activation of apoptotic pathways along with apoptotic cell death [2,5,7]. The tertiary phase occurs following energy failure involving remodeling of the injured brain and cell death [8].

Astrocytes play multiple roles in regulating brain metabolism and serve as gatekeepers of neuronal energy supply [9]. In the presence of oxygen, a high proportion of glucose that enters astrocytes preferentially undergoes glycolysis to produce pyruvate and lactate [10]. Lactate can be used as an energy substrate following its conversion to pyruvate by lactate dehydrogenase 1 in neurons [11]. In addition, astrocytes provide metabolic support to neurons by regulating various pathways, including lipid and glutamate metabolism pathways [11,12].

Astrocytes are affected by hypoxia/ischemia. In a murine model of middle cerebral artery occlusion, it has observed that astrocytes play an essential role in brain ischemic tolerance [13]. To prevent excitotoxicity, astrocytes reduce the levels of excitotoxic amino acids such as glutamate that cause brain damage in HIE by increasing their reuptake [14]. While neurons possess high oxidative metabolism capacity and can easily succumb to ischemia, astrocytes depend more on glycolytic metabolism and thus are less susceptible to oxygen deprivation [15].

We hypothesized that protecting astrocytes following hypoxic–ischemic injury might offer beneficial effects. However, astrocytes’ response to oxygen–glucose deprivation (OGD) is still poorly understood. Herein, we comprehensively analyzed the factors in astrocytes that vary in response to OGD. We identified NR4A as an OGD-responsive factor and performed a detailed analysis of its role in astrocytes.

## 2. Materials and Methods

### 2.1. Animals and Treatment

Pregnant ICR mice were purchased from Charles River Laboratories Japan, Inc. (Yokohama, Japan). All mice were housed in a barrier facility under specific pathogen-free (SPF) conditions and all animal experiments were approved by the Animal Experimentation Ethics Committee of the Tokyo Metropolitan Institute of Medical Science (No. 23-041, approved on 1 April 2023). Animal care was provided in accordance with the institutional animal experimentation guidelines.

### 2.2. Preparation of Astrocyte-Enriched Cultures

Mixed glial culture mainly consists of astrocytes, microglia, and oligodendrocytes. Since hypoxia is known to induce microglial activation, it is important to minimize microglial contamination when studying the role of astrocytes in hypoxic conditions. To deplete microglia, we treated mixed glial cultures with pexidartinib (PLX3397, Cayman Chemical, Ann Arbor, MI, USA), an inhibitor of the colony-stimulating factor-1 receptor (CSF1R), which is critical for microglial survival, and designated these cultures as astrocyte-enriched cultures (AECs). To prepare mixed glial cultures, cerebral cortices from P1–3 newborn ICR mice (3–4 mouse pups were randomly selected, regardless of sex, among the 10–16 littermates) were stripped off meninges and dissociated enzymatically using 0.25% trypsin (Nacalai Tesque, Kyoto, Japan) and 0.04% DNase-I (Roche, Mannheim, Germany), and mechanically by pipetting. Cells were seeded into a 75 cm^2^ flask with Dulbecco’s Modified Eagle Medium (DMEM, Nacalai Tesque) supplemented with 10% fetal bovine serum (FBS) and 1% antibiotics–antimycotics solution (Nacalai Tesque) at a density of 0.8–1 × 10^7^ cells/flask and cultured at 37 °C in a humidified incubator with 5% CO_2_ and 95% air. PLX3397 (10 µM) was added to the medium on days 7 and 9/10 of culture initiation. We previously demonstrated that PLX3397 treatment almost completely removed microglia from mixed glial culture in vitro [16]. After 14 days, the cells were harvested using TrypLE Express (Thermo Fisher Scientific, Waltham, MA, USA) and washed with Imag buffer (PBS with 0.5% bovine serum albumin and 2 mM EDTA). The cells were suspended in DMEM with 10% FBS and seeded at a density of 4–6 × 10^5^ cells/well in a 6-well plate, 2 × 10^4^ cells/well in 96-well plates, or 4 × 10^4^ cells/chamber in Lab-Tek II 8-chamber slides (Nalgene Nunc, Naperville, IL, USA) and further cultured for 5–10 days. Each well was seeded with cells from independent cultures derived from different pups. In total, 18 dams and 219 pups were used in the study.

### 2.3. Oxygen–Glucose Deprivation

Primary AECs were cultured under OGD to mimic hypoxic/ischemic conditions. In brief, cells were plated on 6-well plates, 96-well plates, or 8-chamber slides and cultured in DMEM without glucose (Nacalai Tesque). The cells were then exposed to hypoxia (0–1% O_2_) for 6 or 15 h using the nBIONIX-3 hypoxic cell kit (Sugiyamagen, Tokyo, Japan). The cells along with gas-concentrating agents were placed together in a sealed pouch or tray and incubated at 37 °C for the indicated time. The oxygen concentration was continuously measured using the OXY-2 oxygen monitor (Ichinen Manufacturing, Nagoya, Japan).

### 2.4. Quantitative Real-Time PCR

Total RNA was extracted from untreated or OGD-treated cells using the RNeasy Mini Kit (Qiagen, Hilden, Germany) according to the supplier’s protocol. The quality and concentration of the extracted total RNA were measured using the NanoDrop^TM^ 2000 spectrophotometer (Thermo Fisher Scientific). RNA (600–1000 ng) was used as a template for reverse transcriptase reactions using the PrimeScript^TM^ RT Reagent Kit or Master Mix (Takara Bio, Shiga, Japan). Real-time PCR was performed using 2 µL cDNA, 1 µL forward primer (10 µM), 1 µL reverse primer (10 µM), 12.5 µL TB green Premix EX Taq^TM^ II (Takara Bio), and 8.5 µL Rnase-free distilled water for each sample. The sequences of the primers used in this study are listed in Supplementary Table S1. The thermal cycling conditions were as follows: 30 s at 95 °C followed by 40 cycles of 95 °C for 5 s and 60 °C for 30 s on the Thermal Cycler Dice Real Time System TP870 (Takara Bio). Results are presented as relative expression changes compared to β-actin levels. Data were analyzed by the ΔΔCt method using the Thermal Cycler Dice^®^ Real Time System software Ver.5.1.1 (Takara Bio).

### 2.5. Immunocytochemistry

Primary cells cultured for 3–7 days in Lab-Tek II 8-chamber slides and Lab-Tek 8-chamber permanox slide (Thermo Fisher Scientific) were fixed with 4% paraformaldehyde for 5–10 min and rinsed three times with PBS. The cells were permeabilized by 0.5% Triton-X 100 (Fujifilm Wako Pure Chemicals, Osaka, Japan) for 30 min, rinsed thrice with PBS, and subsequently treated with blocking buffer (Protein Block, Dako, Glostrup, Denmark) for 1 h. Cells were then immunostained with rabbit monoclonal antibodies against HIF-1α (Cell Signaling Technology, Cat# 36169S, RRID: AB_2936488) mouse monoclonal antibodies against GFAP (Cell Signaling Technology, Cat# 3670S, RRID: AB_561049), goat polyclonal antibodies against Iba-1 (Fujifilm Wako Pure Chemicals, Cat# 011-27991, RRID: AB_839504), rabbit polyclonal antibodies against Olig2 (Proteintech, Rosemont, IL, Cat# 13999-1-AP, RRID: AB_2157541), and rabbit polyclonal antibodies against Nr4a1 (Proteintech, Cat# 25851-1-AP, RRID: AB_2880269) overnight at 4 °C. After three washes in PBS-T, cells were incubated with Alexa Fluor 594 goat anti-rabbit IgG (Jackson ImmunoResearch, West Grove, PA, Cat# 111-585-144, RRID: AB_2307325) and Alexa Fluor 488 goat anti-mouse IgG (Jackson ImmunoResearch, Cat# 205-545-108, RRID: AB_2339072) or Alexa Fluor 647 goat anti-mouse IgG (Jackson ImmunoResearch, Cat# 115-605-146, RRID: AB_2338912) for 1 h. Finally, the cells were mounted using Prolong Diamond with DAPI (Thermo Fisher Scientific) and observed using the FV3000 confocal microscope (Olympus, Tokyo, Japan). Mitochondria were labeled using 100 nM MitoTracker Red CMXRos (Thermo Fisher Scientific) for 30 min prior to OGD. Controls included rabbit monoclonal IgG control as an isotype control antibody from the above protocols.

### 2.6. Quantitative Image Analyses

To quantify nuclear fluorescence intensity of HIF-1α or NR4A1, images were captured using the FV3000 confocal microscope and analyzed using ImageJ (NIH, Bethesda, MD, USA, RRID: SCR_003070). Images of four fields at approximately the same location in the well were taken per well at 3× optical magnification with a 20× objective lens. Nuclear areas were determined by DAPI staining and the mean fluorescence intensity of either HIF-1α or NR4A1 was measured in each area using Image J software. An average fluorescence intensity of approximately 30–60 nuclei was calculated per field. Two chambers under identical conditions were evaluated for a single experiment and consequently values from a total of 8 fields were statistically analyzed; the same microscope settings were used in all fields of view. Two investigators (KM and HS) performed the imaging and image analysis separately and in a blinded fashion.

### 2.7. LDH Assay

A commercial lactate dehydrogenase (LDH) kit (Dojindo, Kumamoto, Japan) was used to assess the cytotoxicity according to the manufacturer’s instructions. Briefly, cells were seeded in 96-well plates (1 × 10^4^ cells/well) and either exposed to OGD (6 or 15 h) or left untreated. In some experiments, DIM-C-pPhOH (CDIM8) 20 nM was added 24 h before OGD. The substrate was then added, incubated for 10–20 min, and the stop solution was finally added. LDH activity was determined by measuring absorbance at 490 nm using a plate reader (2030 ARVO X5, PerkinElmer, Waltham, MA, USA). The percentage of dead cells was calculated and represented as the ratio of the LDH activity in the supernatant to total LDH activity. Serum-free DMEM was used during the LDH assay to exclude the effect of LDH present in the FBS.

### 2.8. Transcriptome Analysis

AECs in 6-well plates were either untreated or treated with OGD for 15 h, and total RNA was extracted from cells using the RNeasy Mini kit (Qiagen). The quality of RNA was assessed using the 2100 Bioanalyzer (Agilent Technologies, Santa Clara, CA, USA). After, 200 ng of total RNA was amplified and labeled using the Low Input Quick Amp Labeling Kit (Agilent Technologies) according to the manufacturer’s protocol. Cy3-labeled cRNA was then hybridized to the Mouse Gene Expression 4 × 44 K v2 Microarray (G4846A; Agilent Technologies) using the Gene Expression Hybridization Kit (Agilent Technologies). The slides were washed and scanned using the SureScan Microarray Scanner (Agilent Technologies). The resulting TIFF images were processed using Agilent’s Feature Extraction Software Version 11.5.1.5. Arrays were then 75th-percentile normalized and genes were clustered in GeneSpring GX12.9 (Agilent Technologies) using volcano plots and heatmap analysis. Differentially expressed genes were identified using an unpaired T-test adjusted by the Benjamini–Hochberg method with an adjusted *p*-value cut-off of <0.05 and a fold-change difference of >10. Functional grouping and gene ontology (GO) analysis of differentially expressed genes were performed using DAVID (https://david.ncifcrf.gov/home.jsp (accessed on 4 August 2023)). The dataset generated in this study is available upon reasonable request.

### 2.9. siRNA-Mediated Knockdown

Cells were transfected with siRNAs using Lipofectamine iMAX (Thermo Fisher Scientific). siRNAs targeting *Nr4a1* (#SASI_Mm01_00077215 and #SASI_Mm01_00077216, Sigma-Aldrich, Tokyo, Japan), *Nr4a3* (#SASI_Mm01_00025082 and #SASI_Mm01_00025083, Sigma-Aldrich), and the negative control siRNA (#SIC001, Sigma-Aldrich) were used. Cells were seeded in 6-well plates at a density of 4 × 10^5^ cells/well and transfected with the indicated siRNA at approximately 80–90% confluence, according to the manufacturer’s protocol. Briefly, siRNA oligomers (200 nM) were diluted in 100 µL of Opti-MEM^TM^ I reduced serum medium (Opti-MEM, Thermo Fisher Scientific) and mixed with 6 µL of lipofectamine pre-diluted in 100 µL of Opti-MEM. After 5 min of incubation at room temperature, the mixture (200 µL) was added to each well containing 2 mL of medium. After 24 h of transfection, the medium was replaced with the appropriate medium, and OGD stimulation was performed.

### 2.10. Statistical Analysis

Comparison between the two groups was performed using unpaired two-tailed T-test. Comparison between three or more groups was performed using one-way analysis of variance (ANOVA) followed by Tukey’s post hoc test or Kruskal–Wallis tests followed by the Steel–Dwass post hoc test, as indicated in the text or figure legends. Analyses were performed using EZR [17] and differences were considered significant at *p* < 0.05.

## 3. Results

### 3.1. OGD Induces Cell Death in AECs in a Time-Dependent Manner

**Figure 1:**
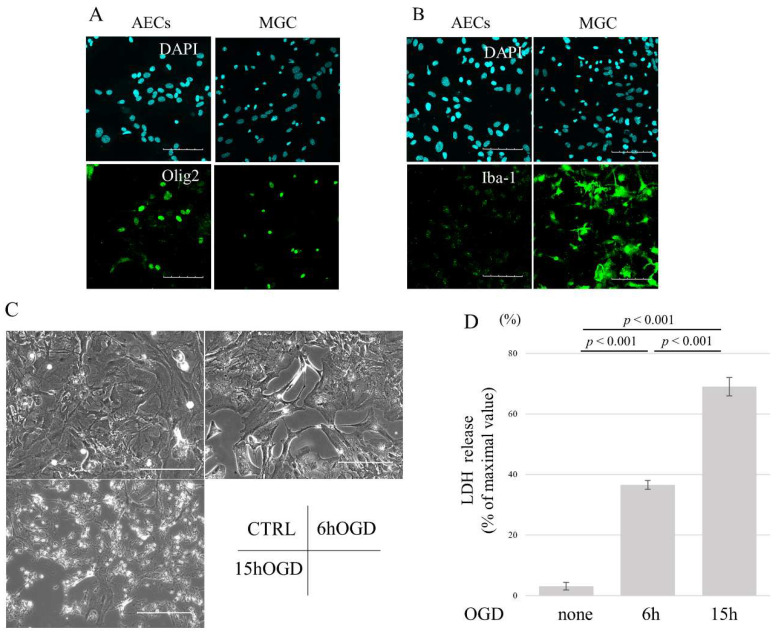
Preparation of astrocyte-enriched culture and oxygen–glucose deprivation (OGD). (**A**) Representative immunofluorescence images showing olig-2-positive cells in mixed glial and astrocyte-enriched cultures. (**B**) Representative immunofluorescence images showing Iba-1-positive cells in mixed glial and astrocyte-enriched cultures. (**C**) Cell morphology after OGD. Control cells were polymorphic and grew in a monolayer, while gaps between cells were observed after 6 h of OGD. After 15 h of OGD, most of the cells retracted their protrusions and became rounded, while some cells were detached. (**D**) Cytotoxicity was evaluated by analyzing LDH release. Cells derived from five independent cultures were used per group. Data are representative of two independent experiments. *p* value is calculated by Kruskal–Wallis with Steel–Dwass post hoc test. Scale bar, 100 µm. CTRL: control, AECs: astrocyte-enriched cultures, MGCs: mixed glial cultures, OGD: oxygen–glucose deprivation, and LDH: lactate dehydrogenase.

Firstly, it was assessed whether microglia were successfully removed from mixed glial cultures following PLX3397 treatment. Immunocytochemistry revealed that while olig-2-positive oligodendrocyte cells were still present, Iba-1-positive microglial cells were almost absent in PLX3397-treated mixed glial cultures (Figure 1A,B). Next, we examined the effects of OGD on astrocyte morphology. While untreated cells formed a sheet-like monolayer, 6 h of OGD resulted in cell body contraction and 15 h of OGD led to marked shrinkage, with some cells being detached (Figure 1C). LDH assay showed that cells under OGD for 6 h released more LDH than untreated cells. LDH release from AECs after 15 h of OGD was greater than that from both untreated cells and cells under OGD for 6 h (Chi-squared = 12.5, df = 2, *p* = 0.024) (Figure 1D). These results indicate that OGD induces cell death in AECs in a time-dependent manner.

### 3.2. OGD Promotes HIF-1α Nuclear Accumulation Leading to the Upregulation of Its Downstream Target Genes in AECs

**Figure 2:**
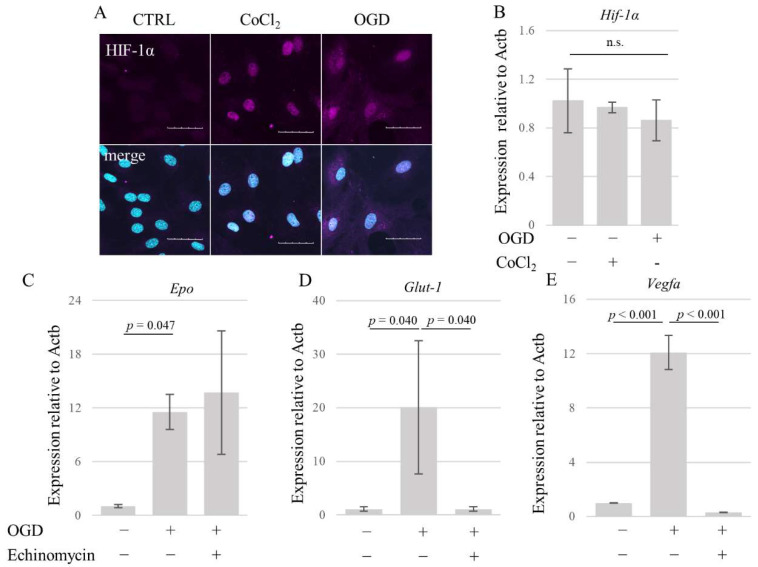
The expression of HIF-1α and downstream target genes in AECs. (**A**) Immunostaining for HIF-1α in unstimulated, CoCl_2_ (500 µM)-stimulated, and OGD (15 h)-stimulated AECs. Scale bar, 50 µm. (**B**) *HIF-1α* mRNA expression in AECs stimulated with 500 µM CoCl_2_ or 15 h OGD evaluated by real-time PCR. (**C**–**E**) mRNA expression of *HIF-1α* downstream genes (**C**) *Epo*, (**D**) *Glut-1*, and (**E**) *Vegfa*) in astrocytes under 15 h of OGD with or without the HIF-1α inhibitor. For (**B**–**E**), mRNA was isolated from the cells derived from three independent cultures, and data are representative of two independent experiments. Error bars represent the mean ± SD. *p* value is calculated by one-way ANOVA with Tukey’s post hoc test. AECs: astrocyte-enriched cultures, CTRL: control, CoCl_2_: cobalt chloride, OGD: oxygen–glucose deprivation, Actb: β-actin, and n.s.: no significance.

Hypoxia-inducible factor-1 alpha (HIF-1α) is a master regulator of oxygen homeostasis. Both cobalt chloride (CoCl_2_) treatment (a known inducer of HIF-1α) and 15 h of OGD induced nuclear expression of HIF-1α (Figure 2A), although no effect on *HIF-1α* mRNA expression was observed (Figure 2B). OGD for 15 h induced the expression of HIF-1α downstream genes including erythropoietin (*Epo)* (F (2, 6) = 8.049, *p* = 0.020, Figure 2C), glucose transporter-1 (*Glut1*) (F (2, 6) = 6.973, *p* = 0.027, Figure 2D), and vascular endothelial growth factor (*Vegfa*) (F (2, 6) = 247.6, *p* < 0.001, Figure 2E). While echinomycin, an inhibitor of HIF-1α, failed to suppress *Epo* expression in AECs exposed to OGD (Figure 2C), it significantly suppressed the expression of *Glut1* (Figure 2D) and *Vegfa* (Figure 2E). These results indicate that 15 h of OGD increases nuclear HIF-1α protein levels, leading to the upregulation of its downstream target genes.

### 3.3. DNA Microarray Identified NR4A1 and NR4A3 as OGD-Responsive Factors

**Figure 3:**
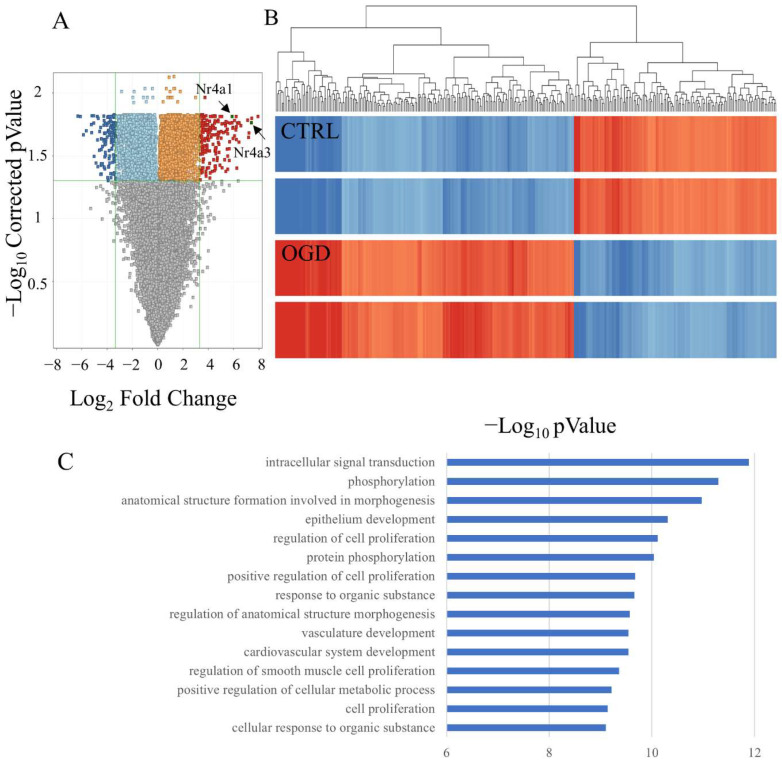
Microarray analysis of AECs treated with OGD. (**A**) Volcano plot shows upregulated (red) and downregulated (blue) genes in AECs cultured under OGD condition. Horizontal axis represents log_2_ fold change and vertical axis represents -log_10_ *p*-value (*p* < 0.05). (**B**) Heatmap shows the overall distribution of differentially expressed genes. Red color represents high expression and blue color represents low expression. (**C**) GO enrichment analysis of differentially expressed genes with top 15 upregulated biological processes based on *p*-values. CTRL: control, AECs: astrocyte-enriched cultures, and OGD: oxygen–glucose deprivation.

To explore the effect of OGD on AECs, we compared gene expression profiles of untreated AECs (*n* = 2) and AECs exposed to OGD for 15 h (*n* = 2) using DNA microarrays. The volcano plot shows that 313 genes were differentially regulated in AECs exposed to 15 h of OGD (188 upregulated and 129 downregulated) compared to the untreated culture (*p* < 0.05; fold change > 10) (Figure 3A). The heatmap shows genes whose expression was elevated following OGD stimulation (Figure 3B). Gene ontology (GO) analysis shows that 160 of GO terms showed significant difference after Bonferroni’s multiple testing correction. These upregulated genes are enriched in GO terms including cell proliferation, intracellular signal transduction, and epithelium development (Figure 3C). The enriched genes showing a significant difference of 0.4 × 10^−5^ or less included the nuclear receptors *Nr4a1* and *Nr4a3* (Table 1). *Nr4a1* and *Nr4a3* were also present in the GO categories anatomical structure formation involved in morphogenesis, positive regulation of cell proliferation, and cellular response to organic substances.

### 3.4. HIF-1α Partly Regulates Nr4a1 and Nr4a3 Expression

**Figure 4:**
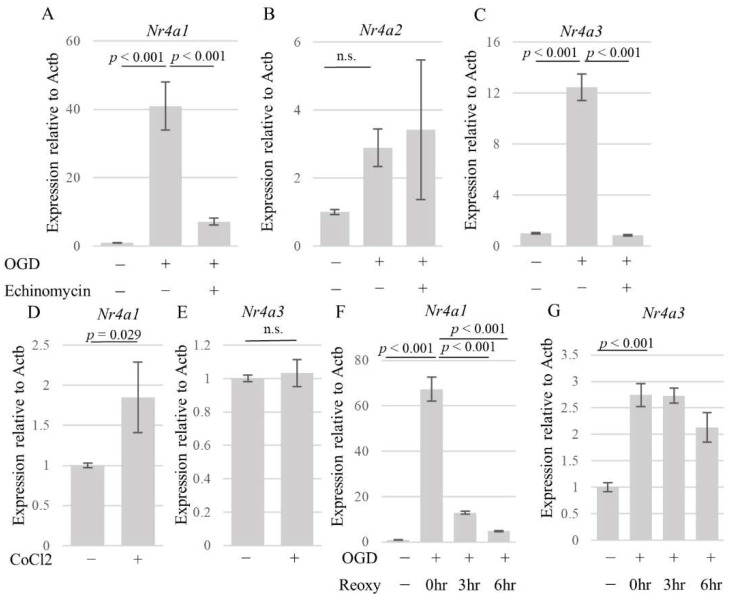
Effects of OGD on the expression of Nr4a mRNAs in AECs. (**A**–**C**) Effects of OGD (15 h) and echinomycin (HIF-1α inhibitor) (20 nM) treatment on the expression of *Nr4a1* (**A**), *Nr4a2* (**B**), and *Nr4a3* (**C**) in AECs. (**D**,**E**) Effects of CoCl_2_ (500 µM) treatment on the expression of *Nr4a1* (**D**) and *Nr4a3* (**E**) in AECs. (**F**,**G**) Time course analysis of (**F**) *Nr4a1* and (**G**) *Nr4a3* in AECs after OGD/reoxygenation. β-actin was used as the housekeeping gene. mRNA was isolated from the cells derived from three independent cultures. Data are representative of two independent experiments. Error bars represent the mean ± SD. *p* value is calculated by one-way ANOVA with Tukey’s post hoc test. OGD: oxygen–glucose deprivation, AECs: astrocyte-enriched cultures, CoCl_2_: cobalt chloride, Reoxy: reoxygenation, ActB: β-actin, and n.s.: no significance.

Our microarray analysis showed that the transcript levels of both *Nr4a1* and *Nr4a3* were upregulated following OGD, suggesting that both of these factors, which are members of the same nuclear receptor group, play essential roles in AECs. First, we validated these results using quantitative real-time PCR. The results showed that the mRNA levels of *Nr4a1*, *Nr4a2*, and *Nr4a3* were upregulated in AECs following OGD (Figure 4A–C). Furthermore, exposure to echinomycin significantly suppressed the expression of *Nr4a1* (F (2, 6) = 82.16, *p* < 0.001) and *Nr4a3* (F (2, 6) = 362.9 *p* < 0.001) in AECs exposed to 15 h of OGD (Figure 4A). Pharmacological induction of HIF-1α using CoCl_2_ increased the expression of *Nr4a1* (Figure 4D) but not *Nr4a3* (Figure 4E). A 3 h OGD also significantly increased the levels of *Nr4a1* (F (3, 8) = 409.9, *p* < 0.001) and *Nr4a3* (F (3, 8) = 52.19, *p* < 0.001) (Figure 4F,G) in AECs. Interestingly, the upregulation of *Nr4a1* persisted for at least 3 h after the termination of OGD (Figure 4F), while *Nr4a3* upregulation persisted for 6 h (Figure 4G).

To investigate genes regulated by NR4A1 and NR4A3 in AECs, we performed knockdown experiments of *Nr4a1*, *Nr4a3*, or both using siRNAs. *Nr4a1* mRNA expression was upregulated with OGD and downregulated in samples transfected with siNr4a1 and those transfected with both siNr4a1 and siNr4a3 (Supplementary Figure S1A). *Nr4a3* mRNA expression was upregulated with OGD and downregulated in samples transfected with siNr4a3 and those transfected with both siNr4a1 and siNr4a3 (Supplementary Figure S1B). We particularly focused on HIF-1α downstream genes, along with genes associated with cell proliferation and inflammation. The results show that except for *Ptgs2* (F (4, 10) = 75, *p* < 0.001) and *Prok2* (F (4, 10) = 65.94, *p* < 0.001) expression, no significant changes were observed in the expression pattern of HIF-1α downstream (HK2, Vegfa), cell proliferation (Adm, Adm2), and immune-associated (Il6, Nfkb1) genes following knockdown (Supplementary Figure S2).

### 3.5. Nr4a1 Translocates from Nucleus to Cytosol following OGD

**Figure 5:**
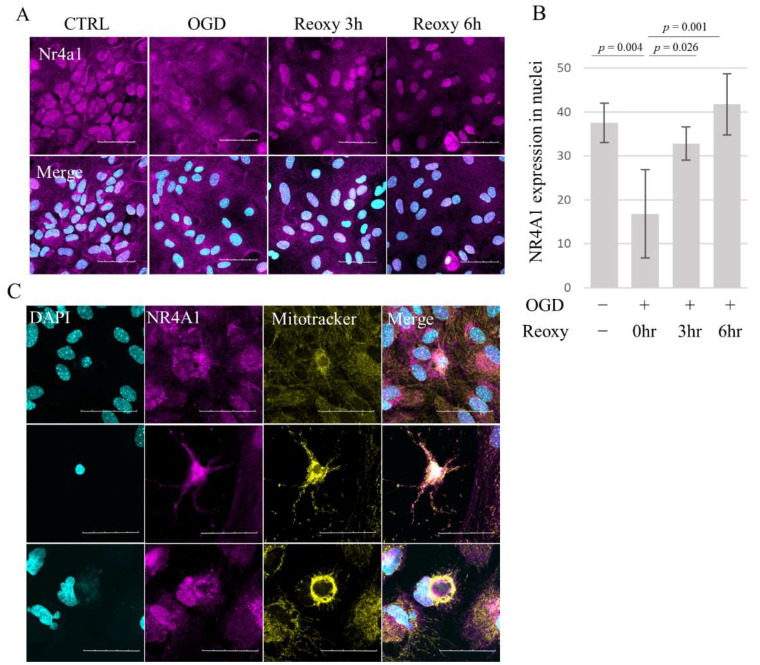
Effects of OGD on subcellular localization of NR4A1 in AECs. (**A**,**B**) Temporal changes in the localization of NR4A1 protein before and after OGD/reoxygenation. Representative immunofluorescence images showing NR4A1 localization merged with DAPI (**A**) and quantitative analysis of NR4A1 expression in the nuclei. Average NR4A1 nuclear expression was isolated from the cells derived from two independent cultures, and the results of the two independent experiments were analyzed cumulatively (*n* = 4 per group) (**B**). (**C**) Co-localization of NR4A1 and mitochondria is more apparent in cells with aggregated nuclei, suggesting that these cells are apoptotic (middle row), and is most prominent in dying cells with disintegrated nuclei (lower row). Scale bar, 100 µm (**A**) and 50 µm (**C**). Error bars represent the mean ± SD. *p* value is calculated by one-way ANOVA with Tukey’s post hoc test. CTRL: control, OGD: oxygen–glucose deprivation, AECs: astrocyte-enriched cultures, and Reoxy: reoxygenation.

We next investigated whether the subcellular localization of the NR4A1 protein is affected by OGD using immunocytochemistry. Before OGD, NR4A1 was localized primarily in the nuclei; however, following OGD, nuclear levels of NR4A1 were significantly reduced (F (3,12) = 10.32, *p* = 0.001) (Figure 5A,B), while cytoplasm levels were increased. Nuclear NR4A1 expression was restored when the cells were returned to the normal culture environment for 3 h after OGD (Figure 5A,B). Co-localization of NR4A1 with the mitochondria was partially observed in presumably apoptotic cells with aggregated nuclei (Figure 5C).

### 3.6. NR4A1 Protects AECs from OGD-Induced Cell Death

**Figure 6:**
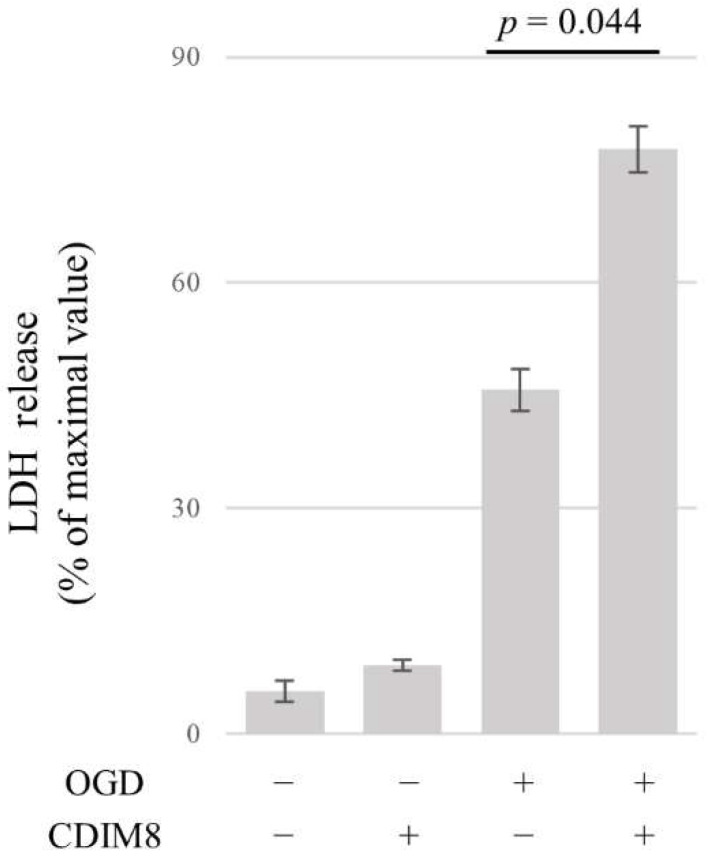
Effects of NR4A1 antagonist on cell death in AECs treated with 6 h of oxygen–glucose deprivation by LDH assay. LDH release from untreated or OGD (6 h)-treated AECs with or without pretreatment (24 h before OGD) with DIM-C-pPhOH (CDIM8, 20 nM), an antagonist of NR4A1. Cells derived from five independent cultures were used per group. Data are shown as a percentage of maximum value in which cells are treated by lysis reagents. Error bars represent the mean ± SD. *p* value is calculated by Kruskal–Wallis with Steel–Dwass post hoc test.

To examine whether Nr4a1 is involved in cell survival, OGD was performed for 6 h after the addition of CDIM8, an antagonist of Nr4a1. The results showed that CDIM8 significantly increased cell death (Chi-squared = 17.857, df = 3, *p* < 0.001) (Figure 6). This indicates that NR4A1 is protective against cell death in an OGD model of AECs.

## 4. Discussion

We investigated the effects of hypoxia/glucose deprivation on astrocytes using the OGD model in AECs and identified NR4A1 and NR4A3 as OGD-inducible factors. The NR4A group of orphan nuclear receptors has three members: NR4A1 (Nur77 or NGFI-B), NR4A2 (Nurr1), and NR4A3 (NOR-1) [18]. The *Nr4a* genes were initially identified as immediate early response genes induced by the nerve growth factor in PC12 cells [19]. NR4As have 90–95% homology in their DNA-binding domains but have divergent N-terminal transcriptional domains. These proteins play key roles in proliferation, apoptosis, neuronal signaling, and hematopoietic, immune, and metabolic processes in many cell types [20].

In regulatory CD4+ T cells (Tregs), NR4A2 directly activates the promoter of the gene encoding the transcription factor Foxp3 [21]. Moreover, *Nr4a* triple deficiency (absence of NR4A1, NR4A2, and NR4A3) aberrantly converts Treg precursors into pathogenic self-reactive cells, suggesting that the NR4A family of receptors plays crucial roles in both Treg cell development and the elimination of cells that fail to mature into Tregs [22]. Increasing evidence suggests that NR4A1 has several metabolic functions in cancers and is involved in regulating glycolysis, fatty acid synthesis, and the metabolism of glutamine and amino acids [23]. NR4A1 and NR4A3 also function as tumor suppressors of myeloid leukemia, and downregulation of NR4A1 and NR4A3 expression is a common feature of the leukemic blast [24]. However, the function of NR4As in hypoxic and ischemic environments has received little attention.

Herein, we found that both *Nr4a1* and *Nr4a3* are regulated by HIF-1α at the mRNA level. In hypoxic conditions, cells respond acutely by inducing adaptive changes in gene expression that either enhance oxygen delivery or promote survival in low-oxygen environments [25]. A pathway mediated by oxygen-dependent post-translational hydroxylation of a transcription factor called hypoxia-inducible factor (HIF) plays an important role in this process [26,27]. HIF is a heterodimer of an unstable α-subunit (HIFα) and a stable β-subunit (HIFβ). Under normoxic conditions, HIFα is polyubiquitinated and degraded via the proteasome, while under hypoxic conditions, the protein is stabilized, leading to its accumulation. The accumulated HIFα forms dimers with the HIFβ subunit and transactivates approximately 100 genes [28]. These genes encode proteins involved in erythropoiesis (EPO), angiogenesis (VEGF), cell survival, and energy metabolism (GLUT-1) [29]. The best-known member of the HIF family is HIF-1α.

HIF-1α binds to the putative HIF-responsive element present in the *Nr4a1* promoter, thereby including NR4A1 expression in renal cell carcinoma [30]. Conversely, exogenously introduced NR4A1 enhances the transcriptional activity of HIF-1 and stabilizes the HIF-1α protein [31]. Hypoxia also upregulates *Nr4a3* transcription via HIF-1α in endothelial cells [32]. However, there are no studies on the effects of hypoxia on NR4A expression in astrocytes. We found that OGD upregulates *Nr4a1* expression and, to a lesser extent, *Nr4a3* expression in AECs via HIF-1α.

Using AECs, we demonstrated that the mRNA levels of *Nr4a1* and *Nr4a3* increase following OGD. However, there was no increase in the protein levels of NR4A1 and NR4A3 immediately after OGD. We hypothesized that this discrepancy between the results at the mRNA and protein levels might be related to changes in the localization of NR4A.

In response to CD437, a retinoic acid receptor γ agonist, NR4A1 translocates from the nucleus to the cytoplasm, where it targets the mitochondria to induce cytochrome c release and apoptosis in LNCaP cells [33].

Mechanistically, NR4A1 targets mitochondria by interacting with Bcl-2, leading to conformational changes in this protein that result in the exposure of its BH3 domain, thereby converting its phenotype to become pro-apoptotic [34]. The present study demonstrated that nuclear levels of NR4A1 were reduced following OGD, while cytoplasmic levels were enhanced in AECs. Furthermore, NR4A1 partially co-localized with mitochondria in cells that were considered to be apoptotic based on morphology. These results indicate that NR4A1 translocates from the nucleus and localizes to mitochondria following OGD in AECs. Considering previous findings that NR4A1 translocation to mitochondria promotes apoptosis, it is possible that NR4A1 translocation is also associated with cell death in astrocytes.

Furthermore, we showed that nuclear NR4A1 levels decreased immediately following OGD and increased again after reoxygenation. We speculate that the increase in *Nr4a1* mRNA expression in response to OGD compensated for the loss of NR4A1 protein expression by cytoplasmic translocation.

Nuclear NR4A1 regulates the expression of several downstream genes. The intriguing pro-apoptotic and growth-inhibitory effects of the NR4A1 antagonist and siNr4a1 were mediated by the downregulation of survivin, a protein that plays a key role in cell cycle progression and inhibits apoptosis by interacting with caspases [35]. Consistently, NR4As are overexpressed in many solid tumor-derived cancer cells compared to their corresponding non-transformed cells [36]. CDIM8 has been reported to inhibit cell growth and promote apoptosis of solid tumor cell lines by acting on NR4A1 and suppressing its production [35,37]. We demonstrated that CDIM8 promoted the cell death of astrocytes exposed to OGD, suggesting that NR4A1 is involved in the survival of astrocytes [38]. Taken together, these results suggest that NR4A1 may be pro-apoptotic when it shuttles out of the nucleus and partially interacts with the mitochondria, whereas in the nucleus, NR4A1 may play a protective role against cell death by regulating the transcription of downstream factors. However, the cytoprotective mechanism of NR4A1 remains unknown, and further studies are needed to investigate whether the nuclear translocation of NR4A1 is affected by its inhibitors.

This study has several limitations. Since the sample size was not calculated before the study, the reliability of the statistical analysis results for some experiments including microarray may be insufficient due to the small sample size. The mixed culture system in our study contained oligodendrocytes and possibly NG2 glia in addition to astrocytes. Therefore, although we showed that NR4A1 and NR4A3 were mainly expressed in astrocytes using immunocytochemistry, the response of oligodendrocytes and NG2 glia to OGD may have affected the results of quantitative PCR and transcriptome analysis. A single housekeeping gene (beta-actin) has been used for real-time PCR study. Although we confirmed that beta-actin gene expression in astrocytes was stable regardless of conditions, gene expression data may need to be reproduced using different housekeeping genes. The expression of the NR4A1 protein was not significantly reduced by siRNA knockdown. We speculate that NR4A1 protein levels may be regulated more strongly by non-transcriptional factors such as protein degradation or changes in localization. We demonstrated that NR4A1 translocates from the nucleus to the cytoplasm where it interacts with mitochondria; however, it remains unclear whether blocking this interaction protects astrocytes from apoptosis. Furthermore, the dynamics of NR4A3 have not been fully characterized and the differential roles of NR4A1 and NR4A3 need further investigation in the future. In addition, it remains to be elucidated whether the response of NR4A1 to OGD and its interaction with HIF-1α occurs similarly in animals exposed to hypoxia and/or ischemia in vivo. It should be considered that astrocytes in vivo, unlike the AEC model, are surrounded by neurons and microglia and are also affected by a variety of molecules, including damage-associated molecular patterns released from dying neurons.

## 5. Conclusions

We demonstrate for the first time that oxygen–glucose deprivation upregulates *Nr4a1* and *Nr4a3* expression in astrocytes, suggesting that these immediate early genes play an essential role in the response to hypoxic and low-glucose stress in astrocytes. Differences between mRNA and protein levels of NR4A1 and the translocation of the NR4A1 protein from the nucleus to the cytoplasm following oxygen–glucose deprivation suggest that NR4A1 plays both genetic and non-genetic roles in astrocytes.

## Figures and Tables

**Table 1:** Upregulated genes in astrocyte-enriched cultures treated with oxygen–glucose deprivation by microarray.

Gene Symbol Name	Fold Change	*p* Value (×10^−5^)
Nfil3	13.3	0.005
Vegfa	11.7	0.010
Runx2	10.9	0.020
Ero1a	22.0	0.030
Lrrfip1	14.1	0.039
Gdf15	11.4	0.043
Errfi1	11.1	0.065
Adm	23.1	0.072
Hk2	11.5	0.074
Trib3	20.3	0.088
Ube2ql1	33.0	0.095
Csf2	19.2	0.107
Areg	17.6	0.107
Ncoa6	22.9	0.109
Kpna7	10.6	0.109
Pvr	12.2	0.109
Prdm10	10.7	0.112
Flt1	11.9	0.118
Tbl2	17.9	0.123
Chac1	22.7	0.143
Ankrd33b	29.9	0.151
Smurf1	10.5	0.155
Fgf21	240.6	0.164
Prok2	73.4	0.165
Nr4a1	58.4	0.167
Tram2	12.8	0.237
Rassf8	14.7	0.277
Egr4	136.0	0.286
Ptgs2	27.1	0.296
Pdxk	11.1	0.302
Amotl1	10.7	0.307
Wdfy1	66.9	0.323
Ereg	11.1	0.336
Skil	22.9	0.340
Tnfaip3	17.0	0.349
Csrnp1	23.1	0.372
Ankrd33b	41.6	0.373
Ern1	27.7	0.375
Atf3	13.3	0.385
Prkd3	44.1	0.391
Adm2	58.8	0.395
Nr4a3	167.6	0.398

## Data Availability

The datasets generated and analyzed in this study are available from the corresponding author upon reasonable request. The data are not publicly available due to privacy and ethical restrictions. Microarray data have been deposited in the National Center for Biotechnology Information Gene Expression Omnibus (GEO; accession number GSE224577).

## Supplementary Materials

The following supporting information can be downloaded at: https://www.mdpi.com/article/10.3390/brainsci14030244/s1, Figure S1: Effects of OGD on AECs transfected with Nr4a1 siRNA, Nr4a3 siRNA, or both siRNA. (A,B) Effects of OGD (6 h) on the expression of Nr4a1 (A) and Nr4a3 (B) in AECs transfected with the indicated siRNA. Figure S2: Effects of OGD on the gene expression in AECs transfected with Nr4a1 siRNA, Nr4a3 siRNA, or both siRNA in AECs. (A–H) Effects of OGD (6 h) on the expression of (A) Vegfa and (B) HK2 (HIF-1α downstream genes), (C) Adm, (D) Adm2, and (E) Prok2 (cell proliferation-associated genes), and (F) Ptgs2, (G) Il-6 and (H) Nfkb1 (inflammation associated genes) in AECs transfected with the Nr4a1 siRNA, Nr4a3 siRNA, or both. Table S1: primers sequences.
