# Supplementary material for: Oxygen–Glucose Deprivation Increases NR4A1 Expression and Promotes Its Extranuclear Translocation in Mouse Astrocytes
Source: Brain Sci. 2024 Feb 29;14(3):244. doi: 10.3390/brainsci14030244 (PMC10968342; doi:10.3390/brainsci14030244)
Supplement: Supplementary file 1 [file brainsci-14-00244-s001.zip › Supplementary.docx]

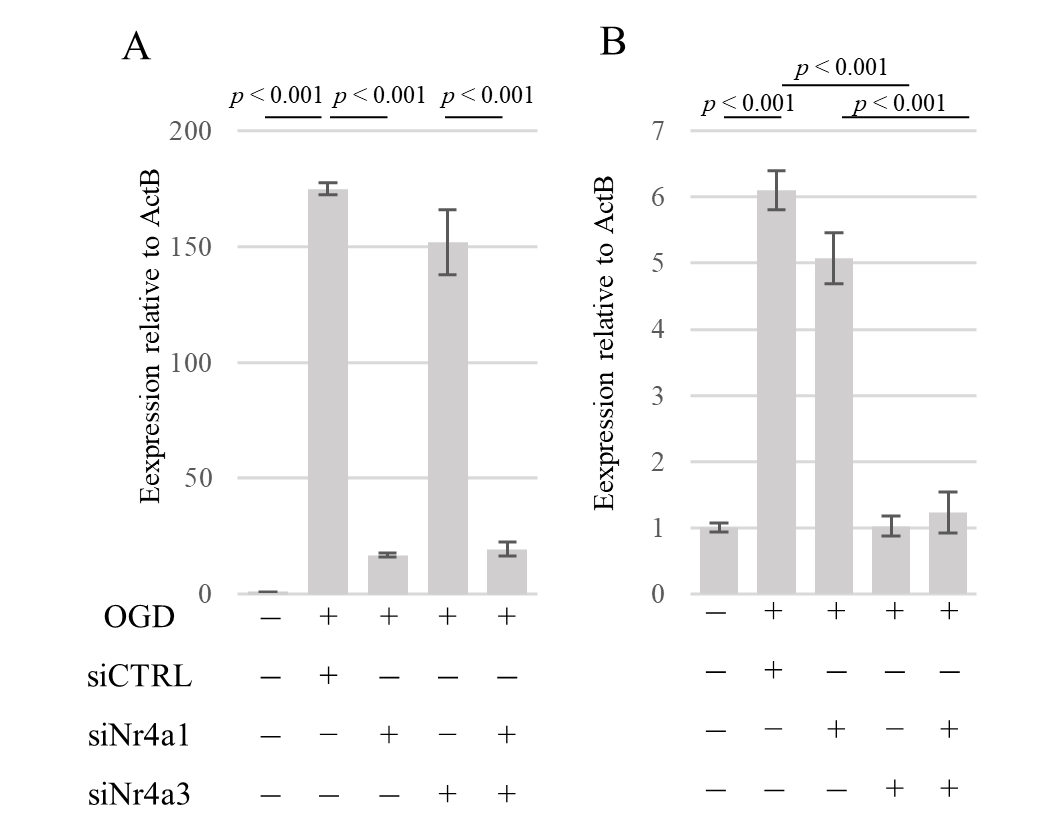


**Figure S1.** Effects of OGD on AECs transfected with Nr4a1 siRNA, Nr4a3 siRNA, or both siRNA. (A and B) Effects of OGD (6 h) on the expression of Nr4a1 (A) and Nr4a3 (B) in AECs transfected with the indicated siRNA. mRNA was isolated from the cells derived from three independent cultures. Data are representative of two independent experiments. P value is calculated by one-way ANOVA with Tukey’s post hoc test. OGD: oxygen-glucose deprivation, AECs: astrocyte-enriched cultures.

**
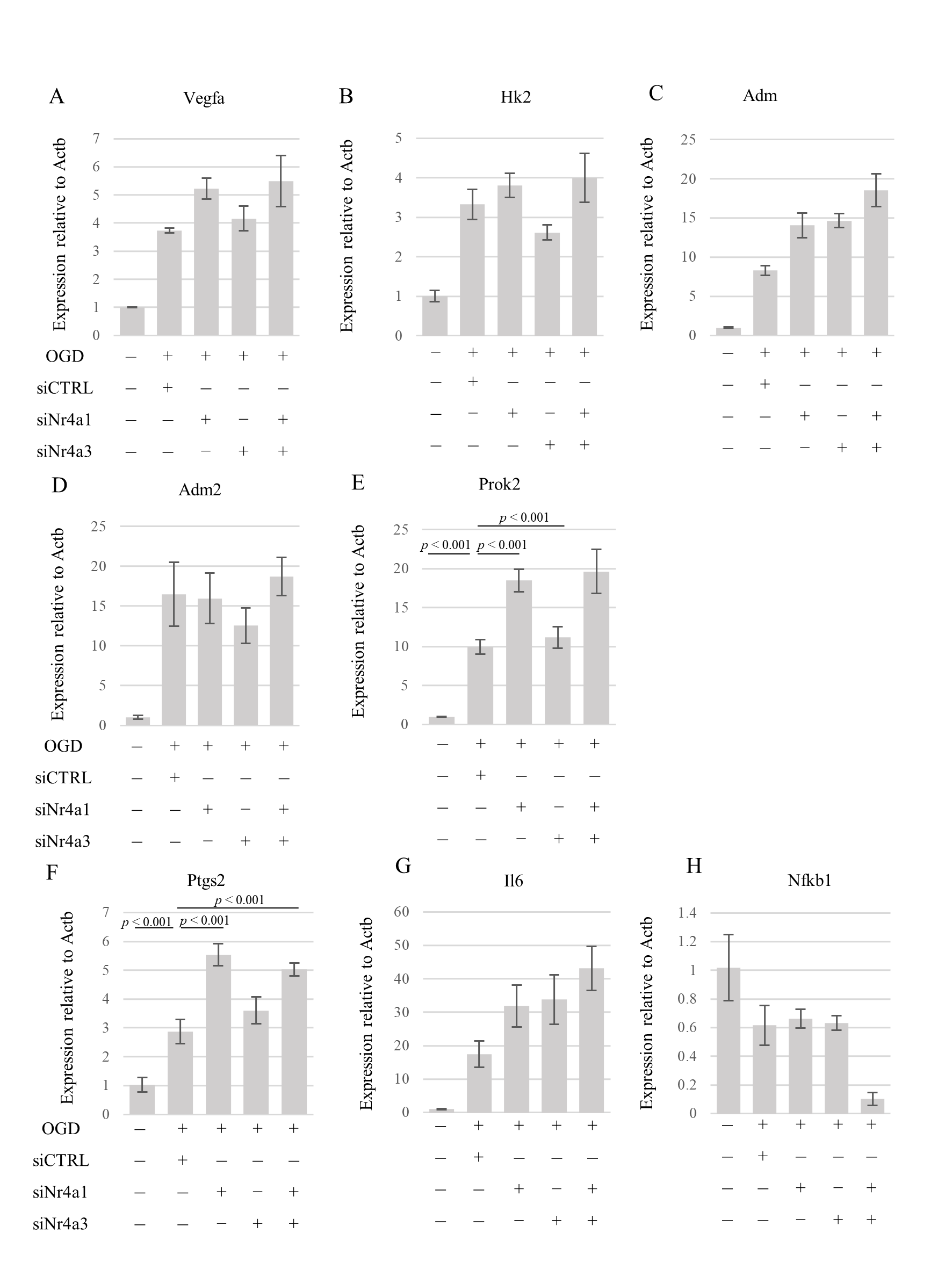
Figure S2.** Effects of OGD on the gene expression in AECs transfected with *Nr4a1* siRNA, *Nr4a3* siRNA, or both siRNA in AECs. (A-H) Effects of OGD (6 h) on the expression of (A) *Vegfa* and (B) *HK2* (HIF-1α downstream genes), (C) *Adm*, (D) *Adm2,* and (E) *Prok2* (cell proliferation-associated genes), and (F) *Ptgs2,* (G) *Il-6* and (H) *Nfkb1* (inflammation associated genes) in AECs transfected with the Nr4a1 siRNA, Nr4a3 siRNA, or both. mRNA was isolated from the cells derived from three independent cultures. Data are representative of two independent experiments. *P* value is calculated by one-way ANOVA with Tukey’s post hoc test. OGD: oxygen-glucose deprivation, AECs: astrocyte-enriched cultures.

**Table S1.** primers sequences.

| Gene | Forward (5’→3’) | Reverse (5’→3’) |
| --- | --- | --- |
| Hif-1α | gcttacacacagaaatggcccag | caccttccacgttgctgacttg |
| Vegfa | ttcgaggaaagggaaagggtcaaa | ctgaacaaggctcacagtgaacg |
| Epo | gcttggaaaagaatggaggtggaag | gtggctgggaggaattggcta |
| Glut-1 | tggtgtcgctgtttgttgta | ctcggccacaatgaaccatg |
| Nr4a1 | cttcttcaagcgcacagtacagaaa | cgtacaacttccttcaccatgcc |
| Nr4a2 | gtgcgtggctttggggaatg | gtctctctgtgaccatagccagg |
| Nr4a3 | caagataccctccagatatgccctg | cttggtgtagtcggggttcatga |
| Adm | cagttcctacccagactcttgatcc | ttcgctctgattgctggcttg |
| Adm2 | agacaacagacgcagccca | cgacgagacttccagactacagg |
| Prok2 | tgctacttctgctgctaccgc | gcactgagagtccttgtcgca |
| Hk2 | taccacacaccctacagcagc | aactcgccatgttctgtccca |
| Il6 | gcaagagacttccatccagttgc | gagtggtatcctctgtgaagtctcc |
| Nfkb1 | aatttgcaactatgtggggcctg | gtgcataccccgtcctcaca |
|  |  |  |
|  |  |  |
|  |  |  |
|  |  |  |
|  |  |  |
|  |  |  |
